# Pediatric and adult glioblastoma radiosensitization induced by PI3K/mTOR inhibition causes early metabolic alterations detected by nuclear magnetic resonance spectroscopy

**DOI:** 10.18632/oncotarget.18206

**Published:** 2017-05-24

**Authors:** Alice Agliano, Geetha Balarajah, Daniela M. Ciobota, Jasmin Sidhu, Paul A. Clarke, Chris Jones, Paul Workman, Martin O. Leach, Nada M.S. Al-Saffar

**Affiliations:** ^1^ Cancer Research UK Cancer Imaging Centre, Division of Radiotherapy and Imaging, The Institute of Cancer Research, London, United Kingdom; ^2^ Cancer Research UK Cancer Therapeutics Unit, The Institute of Cancer Research, London, United Kingdom; ^3^ Divisions of Cancer Therapeutics and Molecular Pathology, The Institute of Cancer Research and The Royal Marsden NHS Foundation Trust, London, United Kingdom; ^4^ The Centre for Molecular Pathology, Division of Cancer Therapeutics, The Institute of Cancer Research, London, United Kingdom

**Keywords:** metabolic biomarker, glioblastoma, PI3K/mTOR, nuclear magnetic resonance spectroscopy, irradiation

## Abstract

Poor outcome for patients with glioblastomas is often associated with radioresistance. PI3K/mTOR pathway deregulation has been correlated with radioresistance; therefore, PI3K/mTOR inhibition could render tumors radiosensitive. In this study, we show that NVP-BEZ235, a dual PI3K/mTOR inhibitor, potentiates the effects of irradiation in both adult and pediatric glioblastoma cell lines, resulting in early metabolic changes detected by nuclear magnetic resonance (NMR) spectroscopy. NVP-BEZ235 radiosensitises cells to X ray exposure, inducing cell death through the inhibition of *CDC25A* and the activation of *p21^cip1^(CDKN1A)*. Lactate and phosphocholine levels, increased with radiation, are decreased after NVP-BEZ235 and combination treatment, suggesting that inhibiting the PI3K/mTOR pathway reverses radiation induced metabolic changes. Importantly, NVP-BEZ235 potentiates the effects of irradiation in a xenograft model of adult glioblastoma, where we observed a decrease in lactate and phosphocholine levels after seven days of combination treatment. Although tumor size was not affected due to the short length of the treatment, a significant increase in *CASP3* mRNA was observed in the combination group. Taken together, our data suggest that NMR metabolites could be used as biomarkers to detect an early response to combination therapy with PI3K/mTOR inhibitors and radiotherapy in adult and pediatric glioblastoma patients.

## INTRODUCTION

Glioblastoma (GBM, WHO grade IV) is the most common primary malignant brain tumor [1]. Patients with newly diagnosed GBM have a median survival of 1 year and generally show a poor response to all therapies [2]. Histologically, pediatric tumors (pGBM) are identical to those arising in adults (aGBM), although the infratentorial region is often affected in children and the supratentorial area is the most common site in adults [1]. Primary aGBM often have activating mutations in receptor tyrosine kinase genes such as EGFR; genes acting in the phosphatidyl inositol 3-kinase (PI3K) pathway; and PTEN [3]. aGBM and pGBM share some DNA abnormalities [1], but mutations in histone H3.3, that occur exclusively in pGBM, have recently been identified [4, 5]. The K27M mutation is typically seen in brainstem or thalamus tumors in younger pediatric patients, while the G34R or G34V mutations are present in adolescent patients with tumors in supratentorial locations [1, 4].

The current standard of care for newly diagnosed GBM is surgery followed by radiotherapy [6]. Radiotherapy was the first adjuvant therapy to demonstrate a significant survival benefit in aGBM [7] and was subsequently also adopted as a standard therapy for pGBM. The administration of temozolomide alongside radiation [6] is currently the standard regimen for newly diagnosed aGBM. This strategy is less effective for pGBM patients, with temozolomide adding only a modest survival benefit [8].

The outcome for patients remains poor despite recent advances in the understanding of GBM biology, developments in surgery, chemotherapy and radiotherapy [1]. To improve patient survival it is likely that current therapies will need to be combined with novel biologically targeted therapies, such as a dual blockade of IGFR1 and PDGFR [9] or mTOR and AKT [10]. Moreover, it has been shown that the radioresistance of glioma cells can in part be attributed to the deregulation of the PI3K/mTOR pathway [11] and that p110_α_ isoform specific PI3K inhibitors increase the cytotoxic effects of radiation therapy in *in vitro* and *in vivo* GBM models [12]. One rationale for investigating the potential utility of PI3K inhibitors as radiosensitising agents is that radiotherapy treatment induces DNA double strand breaks, and PI3K inhibitors can inhibit proteins involved in the DNA damage response. For example, the dual PI3K/mTOR inhibitor NVP-BEZ235 has been shown to be active against ATM serine/threonine kinase (ATM), ATR serine/threonine kinase (ATR), and DNA-PK (DNA protein kinase) [13], independently of the AKT status [14]. The potential of combining irradiation and NVP-BEZ235 to induce tumor growth shrinkage had been previously reported in glioblastoma models (using U87MG cells, [15]), as well as in colorectal [16] and prostate cancer [17].

Identifying biomarkers of target inhibition is critical for assessing the efficacy of treatment in early therapy when tumor shrinkage is not yet detectable, and for correlating antitumor effects with target suppression. The discovery of new imaging biomarkers is playing an increasingly important part in the clinical evaluation of molecular therapeutics, as imaging can provide information about drug distribution and metabolism that otherwise could not be assessed [18]. Non-invasive methods are of particular clinical importance in the study of brain tumors, as GBMs are often inaccessible and hard to biopsy [19, 20]. Nuclear magnetic resonance (NMR) spectroscopy, a powerful tool used to non-invasively detect cell metabolism [21], is capable of measuring a broad range of biological compounds both *in vitro* and *in vivo*, and is useful for characterizing disease and assessing the response to therapy. *In vitro* or *ex vivo* ^1^H-NMR can measure levels of lactate, glutamine, glutamate, creatine, and many other amino acids. It also enables the assessment of choline metabolites, such as phosphocholine (PC) and glycerophosphocholine (GPC) levels [21, 22]. These are associated with the synthesis and catabolism of phosphatidylcholine, contributing to proliferative cell growth [23, 24]. NMR can detect significant differences in the abundance of choline phospholipid metabolites in malignant versus benign lesions in several tumor types [25–27]. The high concentration of PC detected in cancerous tissue has been found to be due, in part, to the stimulation of growth factor-activated Ras and PI3K signaling cascades that increase the expression of choline kinase alpha (CHKA), the enzyme responsible for choline phosphorylation into PC, via the Rho GTPases [28, 29]. In line with this, PI3K inhibition was shown to cause a decrease in PC in breast, prostate and brain cancer cell lines [30–32]. Importantly, lactate and choline metabolites can be assessed non-invasively in clinical settings.

The poor patient outcome, combined with the need for non-invasive biomarkers, encouraged us to investigate whether irradiation potentiation by PI3K/mTOR inhibition with NVP-BEZ235 would result in metabolic changes that can be evaluated by NMR in pediatric and adult GBM models.

Our results confirm previous reports suggesting that inhibition of the PI3K/mTOR pathway increases the cytotoxic activity of irradiation in both pediatric and adult GBM. NMR enables monitoring of metabolic changes occurring during this combination treatment, suggesting that metabolites, such as PC and lactate, may be used as non-invasive biomarkers for the detection of response during early stage clinical trials in patients with glioblastoma.

## RESULTS

### PI3K signaling pathway inhibition enhances cytotoxic X ray irradiation effects in pGBM and aGBM cell lines

**Figure 1:**
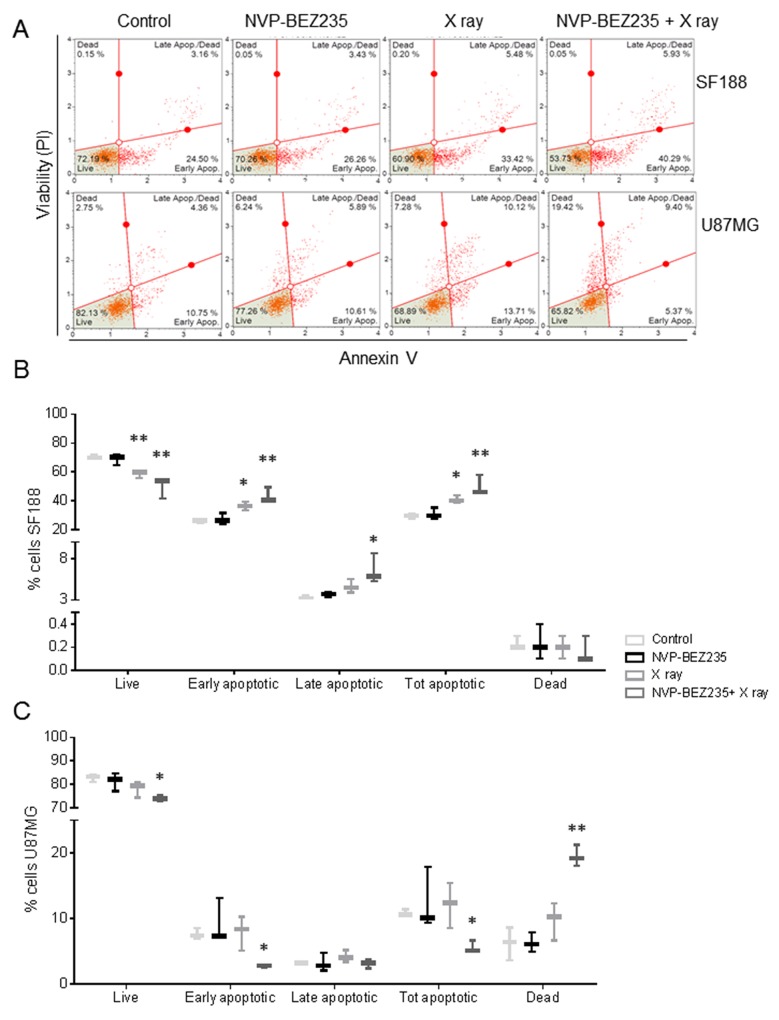
NVP-BEZ235 enhances cytotoxic X ray effects in SF188 and U87MG cell lines **(A)** Representative dot plots showing the response of U87MG and SF188 cell lines to NVP-BEZ235 (2x GI_50_) and X ray (5 Gy). Annexin V staining indicates the apoptotic effect. Gates were positioned according to non-stained cells and negative controls. Percentage of live, apoptotic and dead **(B)** SF188 and **(C)** U87MG cells after treatment with NVP-BEZ235, X rays, alone or in combination (n=3, *P≤0.05, **P≤0.01, ***P≤0.001). SF188 ANOVA test: Live P =0.002; Early apoptotic P =0.0015; Late apoptotic P =0.0151; Tot apoptotic P =0.0015. U87MG ANOVA test: Live P =0.0388; Early apoptotic P =0.0296; Total apoptotic P= 0.048; Dead P= 0.0014.

The pGBM cell line SF188 and the aGBM cell line U87MG were treated with either 5 Gy X ray irradiation, 2x GI_50_ (drug concentration causing 50 % of growth inhibition) NVP-BEZ235 or both treatments in combination for 24 h. The largest decrease in the number of SF188 cells, relative to the untreated control, was observed when they were subject to the combination treatment (viability following combination =63±13 %, P=0.001; viability following NVP-BEZ235 =77±11 %, P=0.006; viability following irradiation =71±9 %; P =0.0002). Treatment action was confirmed by Annexin V staining where the total number of apoptotic cells increased in comparison to the single treatments (Figure 1A and 1B). We also observed that there was a significant decrease in live cells after combination treatment as well as an increase in total apoptotic cells compared to NVP-BEZ235 (p≤ 0.028).

In the U87MG cells, a significant decrease in the number of U87MG cells was only observed following combination treatment (cell viability =88±7 %, P=0.04) compared to control. This decrease in the number of viable cells was associated with an increase of dead cells (20±2 %, P=0.0016), propidium iodide (PI) positive and negative for Annexin V (Figure 1A and 1C). We also observed a significant increase in dead cells following combination treatment relative to both single agents (p≤ 0.002).

**Figure 2:**
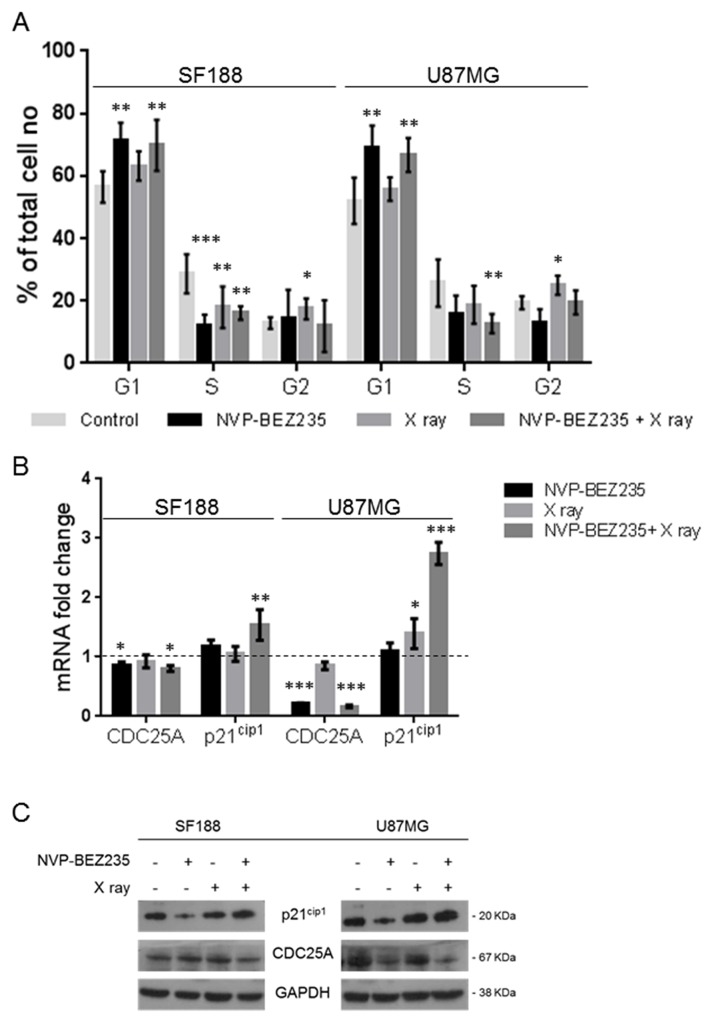
Effects of combination of NVP-BEZ235 and X ray on cell cycle **(A)** Percentage of SF188 and U87MG cells in different phase of the cell cycle following treatment with NVP-BEZ235 and X rays, alone or in combination (n=5, *P≤0.05, **P≤0.01, ***P≤0.001). SF188 ANOVA test: G1 P =0.0017; S P= 0.0001; G2 P= 0.0472. U87MG ANOVA test: G1 P =0.001; S P= 0.0183; G2 P= 0.0038. **(B)** Fold change relative to controls in the levels of *CDC25A* and *p21^Cip1^(CDKN1A)* mRNA after treatment of SF188 and U87MG cell lines with NVP-BEZ235, X ray, alone or in combination (n=3, *P≤0.05, **P≤0.01, ***P≤0.001). SF188 ANOVA test: CDC25A P =0.0283; p21^Cip1^ P= 0.0018. U87MG ANOVA test: CDC25A P =0.0001; p21^Cip1^ P≤ 0.0001. **(C)** Representative immunoblots of CDC25A, p21^Cip1^ in SF188 and U87MG cells following treatment with NVP-BEZ235, X ray or the combination.

Cell cycle analysis (Figure 2A) showed that NVP-BEZ235 treatment induced G1/S arrest (P_SF188_ =0.0023, P_U87MG_=0.0025), while X ray irradiation led to an increase in the G2 phase population (P_SF188_ =0.048, P_U87MG_ =0.042). We also observed that the mRNA expression of cell division cycle 25A (*CDC25A*), a phosphatase required for the progression from G1 to S phase, was also reduced after NVP-BEZ235 treatment (Figure 2B, SF188=0.86, P_SF188_ =0.04; U87MG=0.22, P_U87MG_<0.001), suggesting a possible mechanism for the G1 arrest. As shown in Figure 2A, NVP-BEZ235 treatment was sufficient to reverse the G2/M arrest induced by irradiation in both cell lines (P_SF188_=0.0061, P_U87MG_=0.0075). Q-PCR showed a significant increase in the level of *p21^Cip1^* (*CDKN1A*) mRNA, a potent cyclin-dependent kinase inhibitor protein that regulates cell cycle progression at G1 and S phase (Figure 2B), especially in cells receiving the combination treatment (SF188=1.54, P_SF188_=0.0013; U87MG= 2.75, P_U87MG_<0.0001). For the U87MG cell line such results were also confirmed at protein level by immunoblotting analysis (Figure 2C). For the SF188 cell line changes in protein expression were not evident. This might be due to the smaller differences observed at mRNA level compared to those detected for the U87MG cell line.

### ^1^H-NMR detects metabolic changes after combination treatment with NVP-BEZ235 and X rays in pGMB and aGMB cell lines *in vitro*

**Figure 3:**
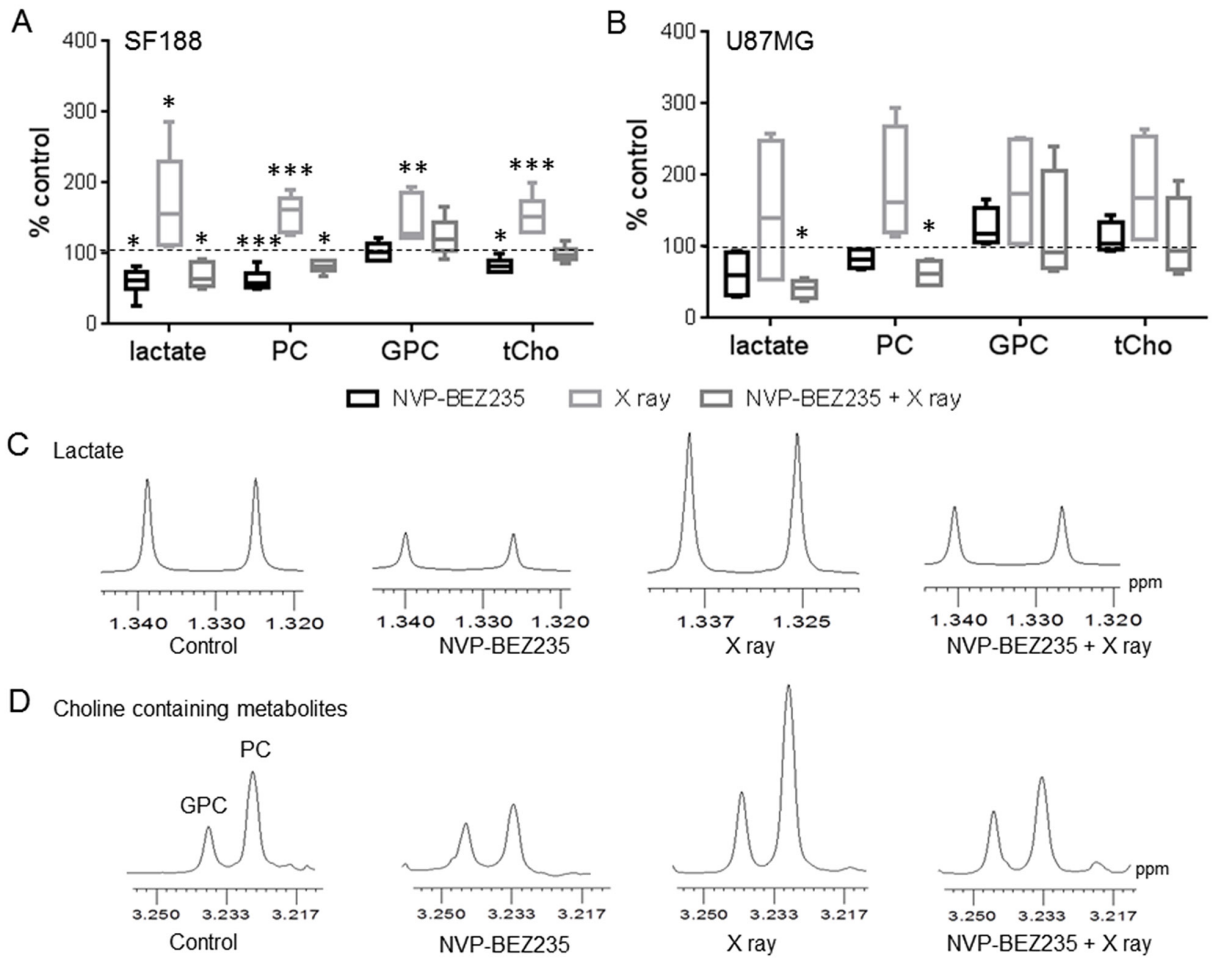
^1^H-NMR metabolic changes after combination treatment with NVP-BEZ235 and X rays Quantitative analysis of metabolic changes detected by ^1^H-NMR following 24 h treatment with NVP-BEZ235, X rays or the combination in **(A)** SF188 and **(B)** U87MG aqueous cell extracts. Data is shown as a percentage of control (n=5, *P≤0.05, **P≤0.01, ***P≤0.001). SF188 ANOVA test: lactate P =0.0008; PC P≤ 0.0001; GPC P= 0.0012; tCho P= 0.0001. U87 ANOVA test: lactate P= 0.031, PC P= 0.0018. Expansion of ^1^H MR spectral regions representing **(C)** lactate and **(D)** choline-containing metabolites in SF188 aqueous cell extracts.

Next, we used ^1^H-NMR to identify potential biomarkers for monitoring the effect of the PI3K/mTOR inhibitor NVP-BEZ235 combined with irradiation on pGMB and aGMB cell lines. ^1^H-NMR aqueous phase analysis of metabolites in the SF188 and U87MG cell lines showed that metabolic changes observed following single agent treatment were still observed after the combination treatment (Figure 3).

Supplementary Figure 1 shows examples of the ^1^H-NMR spectra of control and treated SF188 cells. Treating SF188 cells with NVP-BEZ235 decreased levels of lactate (60±17 %, P=0.042), PC (62±12 %, P=0.0009) and total choline (tCho: 83±10 %, P=0.045) relative to control, but did not affect GPC levels (103±12 %, P=0.58) (Figure 3A). In contrast, X ray exposure increased lactate (162±65 %, P=0.047), PC (157±21 %, P<0.001), GPC (145±28 %, P=0.0021) and tCho (155±28 %, P=0.0001) levels. However, pre-treatment of irradiated cells with NVP-BEZ235 reversed the X ray induced increase in metabolites causing a significant reduction in lactate (69±17 %, P=0.038) and PC (82±8 %, P=0.024) levels. An increase in the level of GPC (130±24 %) was still observed, but it did not reach significance (P=0.08). This, together with the decrease in PC levels, resulted in no significant change in tCho levels (99±11 %, P=0.9) following the combination treatment. Figure 3C and 3D show examples of the lactate and the tCho (PC= GPC= choline) regions of ^1^H-NMR spectra from control, NVP-BEZ235-treated, X ray irradiated and combination-treated SF188 cells. In the U87MG cell line (Supplementary Figure 2 shows the full spectra of control and treated U87MG cells) single treatments proved ineffective but combination of NVP-BEZ235 treatment and X ray irradiation resulted in a significant decrease in lactate (41±11 %, P=0.032) and PC (62±15 %, P=0.045) levels (Figure 3B). Further experiments showed that NVP-BEZ235 administered at a higher concentration equivalent to 5x GI_50_, could induce significant metabolic changes in the U87MG cell line (lactate: 60±26 %, P=0.04; PC: 72±15 %, P=0.03, viability following NVP-BEZ235 5x GI_50_= 86±11 %, P=0.02).

Metabolite expression in conditioned media following treatments has been also quantified. For the SF188 cell line we observed a significant increase in glucose levels following NVP-BEZ235 treatment alone (141.8 ± 35.9%, P= 0.0234) and in combination with X ray (203.1 ± 27.9%, P= 0.0072), lactate levels were decreased without reaching significance. For the U87MG cell line, we observed significant reduction in lactate following both NVP-BEZ235 (76.4± 8.7 %, P= 0.0032) and the combination treatment (79.7± 7.4%, P= 0.0095) in conditioned media. Glucose levels were increased although without reaching significance.

### Enzymes involved in choline and glucose metabolism are altered following inhibition of the PI3K/mTOR pathway

**Figure 4:**
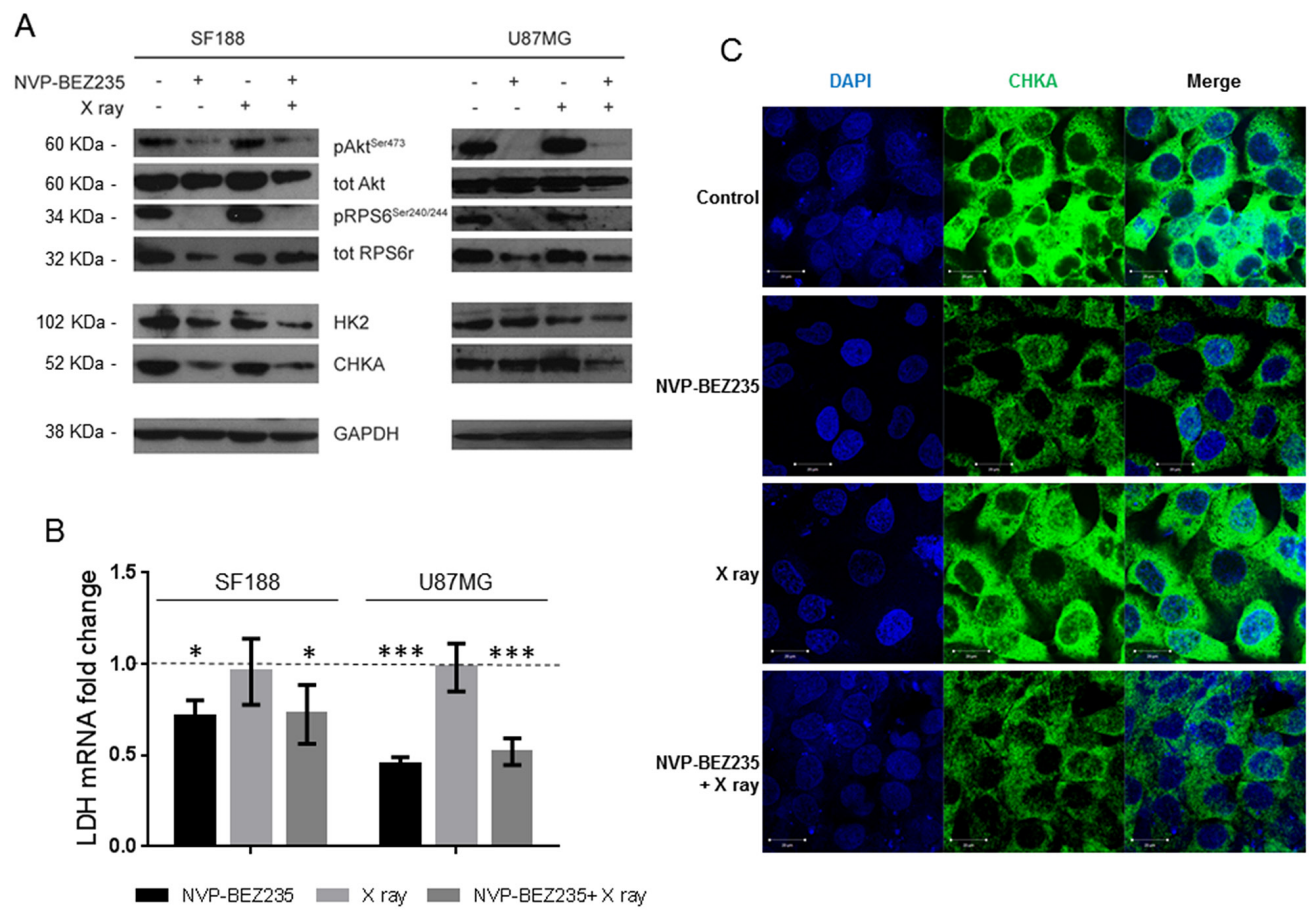
Alteration of enzymes involved in metabolism following treatment with NVP-BEZ235 *in vitro* **(A)** Representative immunoblots of molecular biomarkers from the PI3K/mTOR pathway, HK2, and CHKA, in SF188 and U87MG cells following treatment with NVP-BEZ235, X ray or the combination. **(B)** Fold change in *LDHA* mRNA levels compared to the control after treatment in the SF188 cells and U87MG cells (n=3, *P≤0.05, **P≤0.01, ***P≤0.001). SF188 ANOVA test: P =0.0122; U87MG ANOVA test: P≤ 0.0001. **(C)** Representative immunofluorescence staining for DAPI (blue) and CHKA (green) in SF188 cells before or after treatment with NVP-BEZ235, X ray, alone or in combination (magnification 40X).

In SF188 cells, we observed a significant decrease in the phosphorylation of RPS6 (Ser240/244) following NVP-BEZ235 treatment alone and combined with X ray irradiation (Figure 4A, Supplementary Table 1). In the U87MG cell line we observed a significant decrease in the phosphorylation of AKT (Ser473) and RPS6 (Ser240/244) following combination of NVP-BEZ235 and irradiation (Figure 4A, Supplementary Table 2). Inhibition of the PI3K/mTOR pathway leads to the inhibition of several downstream proteins involved in the metabolic pathway, which might underpin the observed changes in metabolites. In SF188 cells treated with the combination treatment we detected a significant decrease in the amount of the glycolytic enzyme hexokinase II (HK2) and CHKA (Figure 4A, Supplementary Table 1). We also observed a significant decrease in lactate dehydrogenase A (*LDHA*) mRNA (Figure 4B, P<0.037), suggesting inhibition of glycolysis as one of the mechanisms for the depletion of lactate seen in our NMR studies. Following X ray irradiation no changes in the enzymes were observed. In the U87MG cell line, a decrease in the expression levels of CHKA and HK2 was observed only after combination treatment (Figure 4A, Supplementary Table 2). As in SF188 cells, the level of *LDHA* mRNA decreased in the U87MG cell lines after treatment with NVP-BEZ235 alone (P<0.001) or in combination with X rays (Figure 4B, P<0.003).

Immunofluorescence measurements indicated that in the SF188 cell line the decrease in PC observed by NMR after NVP-BEZ235 and combination treatment (Figure 3D) was associated with a decrease in the level of CHKA (Figure 4C). Quantitative analysis of CHKA expression levels by flow cytometry indicated a decrease to 73±5 % (P=0.006) following combination treatment compared to the control, while NVP-BEZ235 treatment reduced the level of CHKA to 76±9 %, without reaching significance (P=0.07). In the U87MG cell line, we observed a decrease in the expression of CHKA by immunoblotting only following combination treatment.

### NMR biomarkers can be identified following NVP-BEZ235 / irradiation combination treatment in tumor extracts of an aGBM model

**Figure 5:**
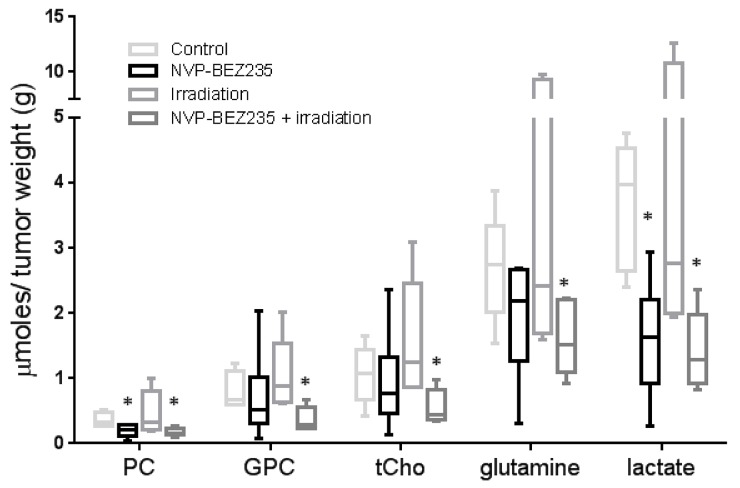
NMR biomarkers after NVP-BEZ235 / irradiation combination treatment in tumor extracts of an aGBM model Quantitative analysis of metabolic changes detected by ^1^H-NMR following treatment of mice bearing U87MG xenografts with NVP-BEZ235, irradiation or the combination (n≥6, *P≤0.05, **P≤0.01, ***P≤0.001). ANOVA test: PC P =0.043; tCho P= 0.042; Lactate P= 0.0083.

**Figure 6:**
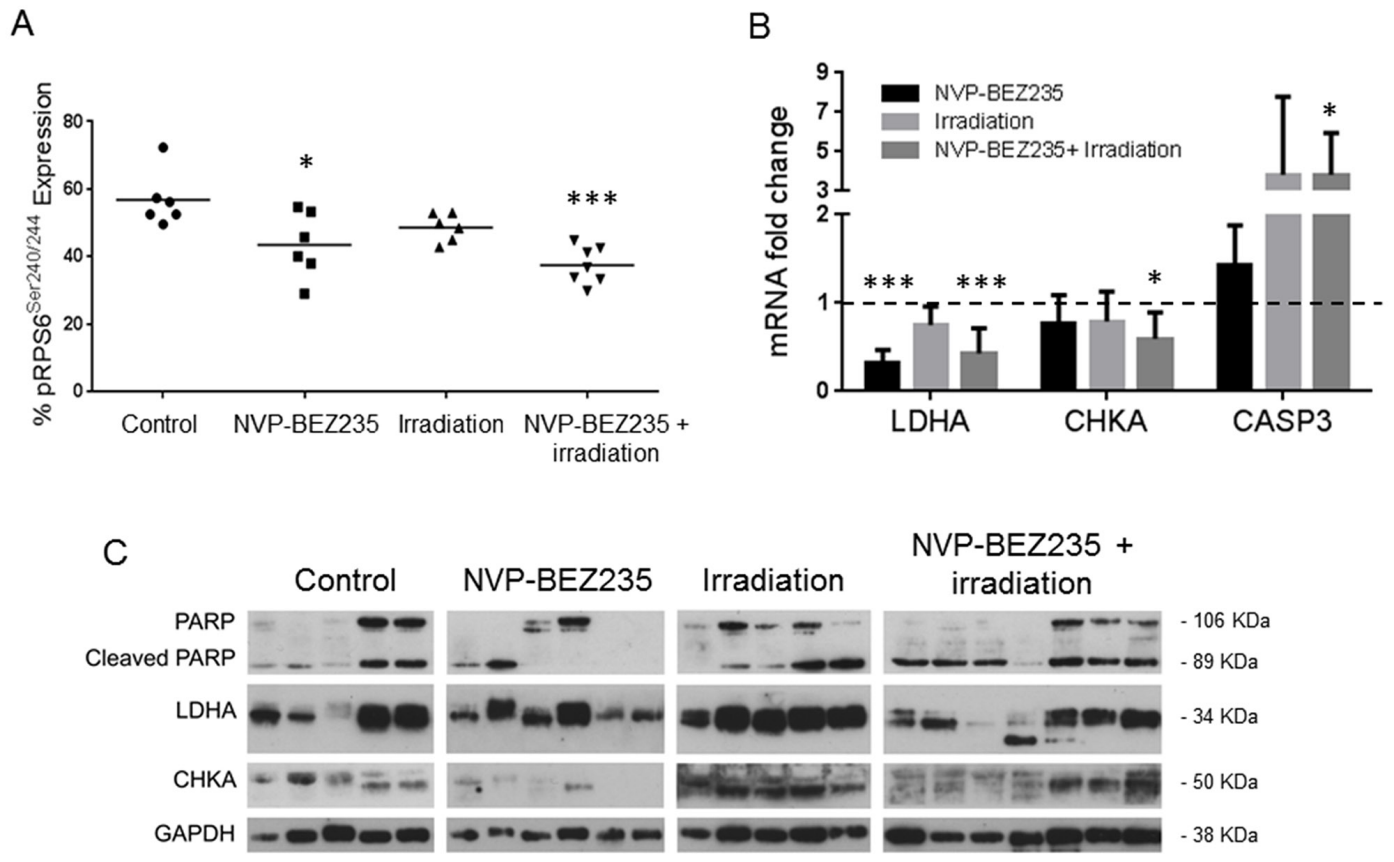
Enzymatic changes involved in metabolism following treatment with NVP-BEZ235 and X ray in tumor extracts of an aGBM model **(A)** MSD analysis for pRPS6^Ser240/244^ in U87MG tumor samples after treatment with NVP-BEZ235, irradiation alone in combination (n≥6, *P≤0.05, **P≤0.01, ***P≤0.001). ANOVA test: P= 0.0008. **(B)** Fold change in levels of *LDHA*, *CHKA* and *CASP3* mRNA after treatment with NVP-BEZ235, irradiation or in combination (n>6, *P≤0.05, **P≤0.01, ***P≤0.001). ANOVA test: LDHA P ≤ 0.0001, CHKA P= 0.048, CASP3 P= 0.043. **(C)** Representative immunoblots showing changes in amounts of PARP/Cleaved PARP, LDHA and CHKA in U87MG xenograft tumors following treatment with NVP-BEZ235, irradiation or the combination (each lane represents a tumor). Tumors were excised at day 8 after treatment.

We further sought to identify whether our *in vitro* biomarker findings could be translated to an *in vivo* model. Since *in vitro* experiments had shown that similar changes in metabolites occurred in the adult U87MG and pediatric SF188 cell lines following combination treatment, we established a mouse model of the U87MG aGBM cells. Treatments did not affect tumor growth within the short time-frame of the experiment but analysis of NMR spectra from *ex vivo* tumor extracts (Supplementary Figure 3A and 3B) showed changes in the levels of metabolites similar to those detected *in vitro*. Treatment with NVP-BEZ235 caused a decrease in the levels of PC (0.2±0.09 μmol/g, P=0.047) and lactate (1.6±0.9 μmol/g, P=0.021) compared with the control (PC: 0.4±0.01 μmol/g, lactate: 3.7±1 μmol/g). Irradiation alone did not induce significant changes in the levels of lactate and PC (P≥0.5). The combination of NVP-BEZ235 with irradiation further decreased the level of lactate (1.4±0.6 μmol/g, P=0.015) and PC (0.17±0.07 μmol/g, P=0.038). Furthermore, we detected a significant decrease in the level of GPC (0.4±0.2 μmol/g, P=0.049), tCho (0.6±0.3 μmol/g, P=0.031) and glutamine (1.6±0.6 μmol/g, P=0.05), compared to the vehicle-treated tumors (GPC: 0.8±0.3 μmol/g, tCho: 1.1±0.5 μmol/g, glutamine: 2.7±0.8 μmol/g). These changes were not observed with the single treatments (Figure 5). A decrease in the percentage of phosphorylated RPS6 (relative to total RPS6 - Ser240/244) was detected by MSD analysis and confirmed inhibition of the PI3K/mTOR pathway (Figure 6A). Q-PCR revealed a significant decrease in the amount of *LDHA* mRNA following both administration of NVP-BEZ235 (P<0.0001) and the combination treatment (P<0.0001). We also detected a significant decrease in *CHKA* mRNA (P=0.05) and an increase in *CASP3* mRNA (P=0.048) after the combination treatment (Figure 6B). Immunoblotting confirmed that there was a significant decrease in the protein expression levels of LDHA (P=0.005) and CHKA (P=0.05) after the combination treatment (Figure 6C, Supplementary Table 3). The expression of CHKA also decreased following administration of NVP-BEZ235 alone. In contrast, tumors treated only with irradiation had increased levels of LDHA (Figure 6C, Supplementary Table 3). Finally, measurements of cleaved PARP showed that the combination of NVP-BEZ235 and irradiation induced significantly more apoptosis than the single treatments (Figure 6C, Supplementary Table 3).

## DISCUSSION

The current standard of care for malignant glioma is initial treatment with radiation therapy combined with temozolomide. However, as GBM usually recur with a median time to progression of approximately 7 months [6], it is clear that more effective therapies are required. Extensive molecular studies have identified several genetic events that occur during glioma formation [33], such as inactivation of the p53 and Rb tumor suppressor pathways and activation of the PI3K pathway. Resistance to radiation has been linked to activation of the PI3K pathway, as activated AKT accelerates repair of the DNA double-strand breaks induced by radiation and, consequently, improves post-irradiation cell survival [34, 35]. Inhibition of the PI3K pathway has been shown to have a radiosensitising effect on tumor cells [14, 36], therefore PI3K inhibitors combined with radiotherapy increase the cytotoxic effect [15]. The results we have presented are consistent with previous findings in aGBM cell lines such as U87MG [14, 15, 37] and in the pGBM cell line SF188 [14] where pretreating with the dual PI3K/mTOR inhibitor NVP-BEZ235 prior to irradiation results in more cell death or apoptosis than the single treatments. Here, we show for the first time that these changes are associated with early detectable metabolic changes that can be monitored by NMR.

We observed that inhibition of the PI3K/mTOR pathway induced cell cycle arrest in G1 phase, in both U87MG adult and SF188 pediatric glioblastoma cell lines. Such arrest can be partly attributed to the inhibition of *CDC25A* and the activation of *p21^Cip1^(CDKN1A)* induced by NVP-BEZ235. CDC25A is a phosphatase required for activation of the G1/S cyclin-dependent kinases CDK4/6, enabling cells to progress from G1 to S phase [38]. p21^Cip1^ (CDKN1A) is a cyclin-dependent kinase inhibitor which promotes G1 cell cycle arrest in response to a variety of cellular stresses [39]. It has been reported that DNA damage, such as that induced by X rays, can promote G1/S and G2/M arrest - with the proportion of cells arresting at G1 or G2 depending on the cell type and the checkpoint controls operating in the cells [40]. In this study, we observed G2/M arrest in both cell lines after irradiation, with *p21^Cip1^ (CDKN1A)* levels particularly increased in U87MG aGBM cells. Such a result is consistent with the fact that G2 phase arrest is known to occur in response to DNA damage, as it blocks the cell's entry into mitosis, and p21^Cip1^ is essential to sustain such arrest [41]. The dual PI3K/mTOR inhibitor NVP-BEZ235 has been reported to be a potent inhibitor of ATM and DNA-PK, two of the major kinases involved in the direct response to DNA double-strands breaks induced by irradiation [13, 14]. By modulating the phosphorylation status of ATM, NVP-BEZ235 radiosensitizes cells, blocking their DNA repair mechanism and forcing them towards apoptosis and death [13, 14]. The combination of NVP-BEZ235 and X ray irradiation induced a G1/S arrest, most likely due to a decrease in CDC25A levels. Surprisingly, levels of *p21^Cip1^(CDKN1A)* RNA were high following combination treatment, suggesting a PI3K-independent regulation of *p21^Cip1^(CDKN1A)* [42]. High levels of *p21^Cip1^ (CDKN1A)*, therefore, are a possible cause of the high mortality observed following the combination treatment, as most of the cells in G1 arrest (>60% of the total population) will continue to apoptosis and death. Moreover, it has been previously reported an enhancement of DNA damage following NVP-BEZ235 treatment as measured by increased expression of histone gamma-H2AX [37].

Our results confirm the potential for combined treatments to improve patient outcome and response to therapy. Currently, patient response to treatment is assessed largely by measuring tumor size by imaging. Although the most common techniques used to measure brain tumors are CT (computerized tomography) and MRI (magnetic resonance imaging), there has been increased interest in the development of imaging techniques that could enable the early detection of a therapeutic response and predict treatment outcome [43]. This is especially true for GBM, where the use of non-invasive methods is of considerable clinical importance because the location of tumors limits accessibility and the repeated use of invasive methods may confer significant risk. In this regard, MR spectroscopy (MRS) can be used to detect metabolic changes that occur early on in a tumor's response to therapy, and can therefore have a role in monitoring treatment outcome facilitating personalized medicine [19, 44, 45]. We have identified lactate and choline metabolites as potential non-invasive biomarkers for monitoring the response to PI3K/mTOR pathway inhibitors, both in pediatric and adult glioblastomas. This supports previous *in vitro* results published by us [30–32] and others [46, 47]. However, we found the U87MG cell line to be less responsive to NVP-BEZ235 than SF188, which may be due to the slower growth rate of U87MG. Further experiments showed that increasing the concentration of NVP-BEZ235 to 5x GI_50_ could induce the same changes in metabolite levels in U87MG cells that were observed in the SF188 cell line. Exposure to radiation induced a significant increase in lactate, PC, GPC and total choline in SF188 cells but not in U87MG cells, probably due to the variation in metabolites levels between different samples masking changes in the U87MG cells. Importantly, the combination of NVP-BEZ235 and radiation induced a decrease in lactate and PC in both cell lines, despite the differences observed in their basal metabolism (Supplementary Table 4), and the effects that radiation has on it. This therefore suggests that our findings might be relevant for both adult and pediatric high grade gliomas independently of their different gene expression patterns [48] and of the presence of specific oncogenic hotspot mutations unique to pGBM [4, 5, 49]. The 2x GI_50_ concentration has been chosen to minimize the cytotoxic side effects and to prove that with low concentration we can observe early metabolic changes by NMR. Cell glycolytic activity was also observed by analyzing the expression of lactate and glucose in conditioned media following treatments. In both cell lines, the combination of NVP-BEZ235 and radiation confirmed the obtained results in the cell extracts. An increase in glucose levels in the media indicates that glycolytic activity is inhibited as cells do not uptake glucose. This results in a decrease in both intracellular and extracellular levels of lactate.

To consider the potential of translating our findings to the clinic, we investigated the effect of combining NVP-BEZ235 treatment and irradiation in an *in vivo* model of adult glioblastoma. Typically, it takes between 2 weeks and 3 months to detect the response of a tumor to treatment in *in vivo* studies [15, 50]. Here, we show changes in metabolite levels can be detected by NMR after only one week of treatment. This demonstrates the potential for NMR to be used to detect the early response of glioblastomas to combination therapy before any variation in tumor size. In the *in vivo* settings a low concentration of NVP-BEZ235 has been used to reduce side effects to undetectable levels. Although tumor size was not affected, a significant increase in *CASP3* mRNA was observed following combination treatment, suggesting increased initiation of apoptosis.

Our *ex vivo* NMR data showed that the combination treatment not only decreased lactate and PC by a similar proportion to NVP-BEZ235 alone, it also decreased glutamine and GPC levels. Moreover, the combined reduction in PC and GPC levels led to a decrease in the levels of total choline. This result is important for the clinical translation of our findings, as a high total choline concentration in untreated tumors is generally associated with tumor malignancy [19, 45, 51]. Over the past decade MRS associated with MRI, has been used routinely in the clinic to aid tumor diagnosis. Proton MRS is able to distinguish the grade and type of primary brain tumor, and inform on grade in prostate and breast malignancies [19, 45, 52]. Another advantage is that spectroscopic alterations are often seen early compared to any variations in tumor size during treatment. Different spectral patterns can be recorded from responding or non-responding patients and can be used to assess the effect of the therapy with the final purpose of tailoring a personalized treatment. Our *ex vivo* data are in line with our *in vitro* results, and clearly show that NMR may be used successfully to detect a decrease in several metabolites, in particular lactate and tCho following inhibition of the PI3K/mTOR pathway and radiation treatment of glioblastoma.

The changes we observed in metabolite concentrations are a consequence of the regulation of cell growth and glucose metabolism by the PI3K/mTOR pathway. Via AKT, PI3K activation stimulates glucose uptake and flux through the early stages of glycolysis, enhancing the expression of glucose transporters and HK2 [22]. In line with this, our *in vitro* experiments showed that levels of the glycolytic enzymes HK2 and LDHA decreased after NVP-BEZ235 treatment combined with irradiation, contributing to the observed reduction in lactate. Furthermore immunoblotting and immunofluorescence analysis showed that the observed decrease in PC was associated with a reduction in the amount of CHKA [30] in response to treatment with NVP-BEZ235 alone and combined with X ray irradiation. Also quantitative FACS analysis confirmed that this reduction is significant following the combination treatment. On the other hand, the increase in lactate and choline metabolites observed following X ray irradiation was not associated with an increase in the protein expression levels of HK2 and LDHA or CHKA, indicating that different mechanisms could be responsible for these changes. PC and GPC increase could be caused by breakdown of cell membranes due cytotoxicity caused by the irradiation, as previously reported [30, 31, 53]. Several papers have described an increase in lactate following irradiation, but as described by Liao *et al*. this might be due to an enhanced LDHA activity rather than an increment in protein expression [54]. Elevated glycolysis and a subsequent elevated production of lactate, as cell response to radiation, might also facilitate DNA double strand break repair [55]. Pre-treatment with NVP-BEZ235 reverses the effects of irradiation, causing metabolic changes similar to those observed with treatment with NVP-BEZ235 alone, indicating that the observed metabolic changes and the associated inhibition of metabolic enzymes are mainly a consequence of the inhibition of the PI3K/mTOR pathway, ruling out non-specific, anti-proliferative effects associated with cytotoxicity.

Our *in vitro* findings are confirmed on excised tumor xenografts by Q-PCR showing a significant reduction in the abundance of the *LDHA* transcript following NVP-BEZ235 and the combination treatment. Furthermore, a decrease in the level of the *CHKA* transcript was detected following the combination treatment. It is important that despite the absence of effects of X ray irradiation on metabolism, it is not interfering with the effects of NVP-BEZ235 and we can still detect the inhibition of enzymes such as LDHA, HK2, CHKA and the associated decrease in lactate and choline metabolites and hence these metabolic changes may have potential as biomarkers for the PI3K pathway inhibition in combination with irradiation.

Taken together, our results show that inhibition of the PI3K/mTOR pathway makes adult and pediatric glioma cells more sensitive to radiation. More importantly, the response to treatment can be detected early by NMR, before tumor size is affected. Treatment induced cell death is associated with changes in the levels of metabolic biomarkers, such as lactate and choline, that could predict tumor response. To our knowledge, this is the first study showing that a PI3K inhibitor combined with radiation induces metabolic changes in adult and pediatric glioblastoma cells. In the clinical setting, the metabolites we have identified here may have potential for use as non-invasive biomarkers to detect a tumor response prior to detecting any changes in tumor size. In turn, this should enable clinicians to personalize the treatment of adult and pediatric patients affected by glioma, which at the moment is a disease with a very poor prognosis.

## MATERIALS AND METHODS

### Cell culture and treatment

The human adult U87MG (*PTEN* null) and pediatric SF188 (WT *PTEN*, *PIK3CA and* histone H3.3 (*H3F3A*) *G34V*) glioblastoma cell lines (WHO grade IV) were grown as monolayers in DMEM = Glutamax (Gibco, Carlsbad, CA, USA) and DMEM/F12 Ham's (Sigma, St. Louis, MO, USA) medium respectively, supplemented with 10% FCS (fetal calf serum, Gibco, Carlsbad, CA, USA) and maintained at 5% CO_2_. Both cell lines have been kindly donated by Dr. C. Jones and authenticated by STR profiling (02/2015 for the U87MG, and 03/2016 for the SF188). Cell viability was judged by trypan blue exclusion.

The GI_50_ value for the dual pan-Class I PI3K/mTOR inhibitor NVP-BEZ235 (Novartis, Basel, Switzerland) was determined using a cell titre blue assay (Promega, Madison, WI, USA). Both cell lines were treated with 2x GI_50_ NVP-BEZ235 as a single treatment (U87MG GI_50_ = 35.7nM, SF188 GI_50_ = 14nM) for 24 h. When given as part of a combined treatment, NVP-BEZ235 (2x GI_50_) was administered 1 h prior to X ray irradiation (5 Gy, AGO X ray HS 320Kv set, AGO Installations Ltd) then maintained for 24 h. The effect of treatment on cell number was monitored by counting the number of viable attached cells in a treated flask and comparing that number with the number of attached cells in a control flask. Cell viability was evaluated using the Annexin V & Cell death Assay (Muse, Millipore, Billerica, MA, USA) following the manufacturer's instructions.

### Flow cytometry

Cell cycle analysis was performed as previously described [56]. Acquisitions were performed using a BD LSRII flow cytometer. Cell cycle data were analyzed using FlowJo software (Ashland).

### Xenograft studies

All experimental procedures on animals were carried out in accordance with U.K. Home Office regulations under the Animals (Scientific Procedures) Act 1986 and UK National Cancer Research Institute (NCRI) Guidelines for the Welfare and Use of Animals in Cancer Research [57]. U87MG cells (2x 10^6^ in 200 μl of HBSS, Hanks' Balanced Salt Solution, Gibco, Carlsbad, CA, USA) were delivered subcutaneously into athymic nude mice, NCr-*Foxn1^nu^* (female, 6 weeks old, 20g, Taconic, Albany, NY, USA). When tumors reached a volume of 200 mm^3^, mice were randomly divided into four cohorts: vehicle, NVP-BEZ235, irradiation, and combined NVP-BEZ235 = irradiation treatment. NVP-BEZ235 (45 mg/kg) or vehicle administration (N-methyl-2-pyrrolidone combined with Polyethylene glycol, Sigma, St. Louis, MO, USA) was given p.o. on days 1, 3, 5 and 7. Irradiation (2 Gy/dose) was given on days 2, 4 and 6 using a single Caesium137 source (Gammacel 40 Exactor, Best Teratonic). Animals were euthanized at day 8 and tumors were snap-frozen in liquid nitrogen for *ex vivo* NMR and immunoblotting analysis.

### Immunoblotting and MSD analysis

Immunodetection was performed using antibodies against pAKT (Ser473), total AKT, pRPS6 (Ser240/244), total RPS6, hexokinase II (HK2, Cell Signaling, Beverly, MA, USA), CHKA (Sigma), p21^Cip1^ (Cell Signaling), Cdc25A (Santa Cruz Biotechnology, Santa Cruz, CA, USA) and GAPDH (Chemicon, Billerica, MA, USA). Blots were revealed with peroxidase-conjugated secondary anti-rabbit antibody (GE Healthcare, Chalfont St. Giles, UK) followed by ECL chemiluminescence solution (Amersham Biosciences, St. Giles, UK). The western blot procedure used for the tumor extracts was the same used for the cell lines.

An MSD assay, more sensitive than immunoblots with tissue extracts, was used to identify target inhibition *ex vivo* in tissue samples. It was based on a previously described protocol with minor modifications [58]. The protein concentration was determined using a Direct Detect spectrophotometer according to the manufacturer's instructions.

### RNA extraction and RT-PCR

Total cellular RNA was extracted using a QIAamp RNeasy mini extraction kit (Qiagen, Hilden, Germany). Tumor RNA was extracted using a RecoverAll™ Total Nucleic Acid Isolation Kit for FFPE (Ambion, Foster City, CA, USA). To generate cDNA, 1000 ng of total RNA was reverse transcribed using a high capacity cDNA reverse transcription kit (Applied BioSystems, Carlsbad, CA, USA). Real-time RT-PCR analysis was performed to quantify *LDHA* (Hs00855332_g1), *CDC25A* (Hs00947994_m1), *p21^cip1^(CDKN1A)* (Hs00355782_m1), *CASP3* (Hs00234387_m1) and *CHKA* (Hs00957875_m1) mRNA using a TaqMan fast advanced master mix (Applied BioSystems, Carlsbad, CA, USA) following manufacturer's instructions.

### ^1^H-NMR of cell and tumor extracts

To obtain an NMR spectrum, an average of 3×10^7^ cells in logarithmic phase was extracted from cell culture using the dual phase extraction method [56]. Snap-frozen excised tumors had been reduced to a cell suspension and were then treated as cell culture samples. Lyophilized samples of the water soluble fraction were reconstituted in deuterium oxide (D_2_O). ^1^H-NMR spectra were acquired as previously described [56]. Metabolite contents were determined by integration and normalized relative to the peak integral of an internal reference (TSP 0.15%) and corrected for signal intensity saturation and the number of cells extracted per sample, or the tumor weight.

### Immunofluorescence (IF)

Cells, grown on glass coverslips in 24 well plates and treated as described above, were fixed in 4% paraformaldehyde, permeabilized with Triton and incubated with primary (CHKA, Sigma, St. Louis, MO, USA) and secondary antibody (anti-Rabbit, Alexa Fluor 488, Cell Signaling, Beverly, MA, USA) for 1 h each at 4°C. Images were captured with a Zeiss confocal microscope LSM700 and analyzed with the Zen2009 software.

### Statistical analysis

Box and whiskers plots divide the data set into quartiles to display sample variability and degree of dispersion (line at median) ± S.D. Histograms are expressed as mean ± S.D. One way ANOVA test was used to verify the statistical significance of the results, with a p-value of ≤ 0.05 considered to be significant. All experiments are n ≥ 3.

## SUPPLEMENTARY MATERIALS FIGURES AND TABLES











## Abbreviations

aGBM: adults GBM
ATM: ataxia telangiectasia mutated serine/threonine kinase
ATR: ataxia telangiectasia and Rad3-related protein serine/threonine kinase
CDC25A: cell division cycle 25A
CHKA: choline kinase alpha
CT: computerised tomography
DNA-PK: DNA protein kinase
FACS: fluorescence activated cell sorting
FCS: fetal calf serum
GBM: glioblastoma multiforme
GI50: 50 % of growth inhibition
GPC: glycerophosphocholine
HBSS: Hanks' balanced salt solution
HK2: hexokinase II
LDHA: lactate dehydrogenase A
MRI: magnetic resonance imaging
MRS: MR spectroscopy
MSD: Meso Scale Discovery
NMR: nuclear magnetic resonance
PC: phosphocholine
pGBM: pediatric GBM
PI3K: phosphatidyl inositol 3-kinase
tCho: total choline.

## References

[1] Jones C, Perryman L, Hargrave D (2012). Paediatric and adult malignant glioma: close relatives or distant cousins?. Nat Rev Clin Oncol.

[2] Mischel PS, Cloughesy TF (2003). Targeted molecular therapy of GBM. Brain Pathol.

[3] Furnari FB, Fenton T, Bachoo RM, Mukasa A, Stommel JM, Stegh A, Hahn WC, Ligon KL, Louis DN, Brennan C, Chin L, DePinho RA, Cavenee WK (2007). Malignant astrocytic glioma: genetics, biology, and paths to treatment. Genes Dev.

[4] Schwartzentruber J, Korshunov A, Liu XY, Jones DT, Pfaff E, Jacob K, Sturm D, Fontebasso AM, Quang DA, Tonjes M, Hovestadt V, Albrecht S, Kool M (2012). Driver mutations in histone H3.3 and chromatin remodelling genes in paediatric glioblastoma. Nature.

[5] Bjerke L, Mackay A, Nandhabalan M, Burford A, Jury A, Popov S, Bax DA, Carvalho D, Taylor KR, Vinci M, Bajrami I, McGonnell IM, Lord CJ (2013). Histone H3.3 mutations drive pediatric glioblastoma through upregulation of MYCN. Cancer Discov.

[6] Stupp R, Hegi ME, Mason WP, van den Bent MJ, Taphoorn MJ, Janzer RC, Ludwin SK, Allgeier A, Fisher B, Belanger K, Hau P, Brandes AA, Gijtenbeek J (2009). Effects of radiotherapy with concomitant and adjuvant temozolomide versus radiotherapy alone on survival in glioblastoma in a randomised phase III study: 5-year analysis of the EORTC-NCIC trial. Lancet Oncol.

[7] Laperriere N, Zuraw L, Cairncross G, Cancer Care Ontario Practice Guidelines Initiative Neuro-Oncology Disease Site Group (2002). Radiotherapy for newly diagnosed malignant glioma in adults: a systematic review. Radiother Oncol.

[8] Ruggiero A, Cefalo G, Garre ML, Massimino M, Colosimo C, Attina G, Lazzareschi I, Maurizi P, Ridola V, Mazzarella G, Caldarelli M, Di Rocco C, Madon E (2006). Phase II trial of temozolomide in children with recurrent high-grade glioma. J Neurooncol.

[9] Szerlip NJ, Pedraza A, Chakravarty D, Azim M, McGuire J, Fang Y, Ozawa T, Holland EC, Huse JT, Jhanwar S, Leversha MA, Mikkelsen T, Brennan CW (2012). Intratumoral heterogeneity of receptor tyrosine kinases EGFR and PDGFRA amplification in glioblastoma defines subpopulations with distinct growth factor response. Proc Natl Acad Sci U S A.

[10] Little SE, Popov S, Jury A, Bax DA, Doey L, Al-Sarraj S, Jurgensmeier JM, Jones C (2012). Receptor tyrosine kinase genes amplified in glioblastoma exhibit a mutual exclusivity in variable proportions reflective of individual tumor heterogeneity. Cancer Res.

[11] Begg AC, Stewart FA, Vens C (2011). Strategies to improve radiotherapy with targeted drugs. Nat Rev Cancer.

[12] Chen JS, Zhou LJ, Entin-Meer M, Yang X, Donker M, Knight ZA, Weiss W, Shokat KM, Haas-Kogan D, Stokoe D (2008). Characterization of structurally distinct, isoform-selective phosphoinositide 3'-kinase inhibitors in combination with radiation in the treatment of glioblastoma. Mol Cancer Ther.

[13] Toledo LI, Murga M, Zur R, Soria R, Rodriguez A, Martinez S, Oyarzabal J, Pastor J, Bischoff JR, Fernandez-Capetillo O (2011). A cell-based screen identifies ATR inhibitors with synthetic lethal properties for cancer-associated mutations. Nat Struct Mol Biol.

[14] Mukherjee B, Tomimatsu N, Amancherla K, Camacho CV, Pichamoorthy N, Burma S (2012). The dual PI3K/mTOR inhibitor NVP-BEZ235 is a potent inhibitor of ATM- and DNA-PKCs-mediated DNA damage responses. Neoplasia.

[15] Gil del Alcazar CR, Hardebeck MC, Mukherjee B, Tomimatsu N, Gao X, Yan J, Xie XJ, Bachoo R, Li L, Habib AA, Burma S (2014). Inhibition of DNA double-strand break repair by the dual PI3K/mTOR inhibitor NVP-BEZ235 as a strategy for radiosensitization of glioblastoma. Clin Cancer Res.

[16] Chen YH, Wei MF, Wang CW, Lee HW, Pan SL, Gao M, Kuo SH, Cheng AL, Teng CM (2015). Dual phosphoinositide 3-kinase/mammalian target of rapamycin inhibitor is an effective radiosensitizer for colorectal cancer. Cancer Lett.

[17] Potiron VA, Abderrahmani R, Giang E, Chiavassa S, Di Tomaso E, Maira SM, Paris F, Supiot S (2013). Radiosensitization of prostate cancer cells by the dual PI3K/mTOR inhibitor BEZ235 under normoxic and hypoxic conditions. Radiother Oncol.

[18] Workman P, Aboagye EO, Chung YL, Griffiths JR, Hart R, Leach MO, Maxwell RJ, McSheehy PM, Price PM, J; Zweit, Cancer Research UK Pharmacodynamic/Pharmacokinetic Technologies Advisory Committee (2006). Minimally invasive pharmacokinetic and pharmacodynamic technologies in hypothesis-testing clinical trials of innovative therapies. J Natl Cancer Inst.

[19] Peet AC, Arvanitis TN, Leach MO, Waldman AD (2012). Functional imaging in adult and paediatric brain tumours. Nat Rev Clin Oncol.

[20] Li Y, Lupo JM, Parvataneni R, Lamborn KR, Cha S, Chang SM, Nelson SJ (2013). Survival analysis in patients with newly diagnosed glioblastoma using pre- and postradiotherapy MR spectroscopic imaging. Neuro Oncol.

[21] Beloueche-Babari M, Chung YL, Al-Saffar NM, Falck-Miniotis M, Leach MO (2010). Metabolic assessment of the action of targeted cancer therapeutics using magnetic resonance spectroscopy. Br J Cancer.

[22] Vander Heiden MG, Cantley LC, Thompson CB (2009). Understanding the Warburg effect: the metabolic requirements of cell proliferation. Science.

[23] Aboagye EO, Bhujwalla ZM (1999). Malignant transformation alters membrane choline phospholipid metabolism of human mammary epithelial cells. Cancer Res.

[24] Baek HM, Chen JH, Nalcioglu O, Su MY (2008). Choline as a biomarker for cell proliferation: do the results from proton MR spectroscopy show difference between HER2/neu positive and negative breast cancers?. Int J Cancer.

[25] Glunde K, Jie C, Bhujwalla ZM (2004). Molecular causes of the aberrant choline phospholipid metabolism in breast cancer. Cancer Res.

[26] Horska A, Barker PB (2010). Imaging of brain tumors: MR spectroscopy and metabolic imaging. Neuroimaging Clin N Am.

[27] Kurhanewicz J, Swanson MG, Nelson SJ, Vigneron DB (2002). Combined magnetic resonance imaging and spectroscopic imaging approach to molecular imaging of prostate cancer. J Magn Reson Imaging.

[28] Ramirez de Molina A, Penalva V, Lucas L, Lacal JC (2002). Regulation of choline kinase activity by Ras proteins involves Ral-GDS and PI3K. Oncogene.

[29] Ramirez de Molina A, Rodriguez-Gonzalez A, Gutierrez R, Martinez-Pineiro L, Sanchez J, Bonilla F, Rosell R, Lacal J (2002). Overexpression of choline kinase is a frequent feature in human tumor-derived cell lines and in lung, prostate, and colorectal human cancers. Biochem Biophys Res Commun.

[30] Al-Saffar NM, Jackson LE, Raynaud FI, Clarke PA, Ramirez de Molina A, Lacal JC, Workman P, Leach MO (2010). The phosphoinositide 3-kinase inhibitor PI-103 downregulates choline kinase alpha leading to phosphocholine and total choline decrease detected by magnetic resonance spectroscopy. Cancer Res.

[31] Al-Saffar NM, Marshall LV, Jackson LE, Balarajah G, Eykyn TR, Agliano A, Clarke PA, Jones C, Workman P, Pearson AD, Leach MO (2014). Lactate and choline metabolites detected in vitro by nuclear magnetic resonance spectroscopy are potential metabolic biomarkers for PI3K inhibition in pediatric glioblastoma. PLoS One.

[32] Beloueche-Babari M, Jackson LE, Al-Saffar NM, Eccles SA, Raynaud FI, Workman P, Leach MO, Ronen SM (2006). Identification of magnetic resonance detectable metabolic changes associated with inhibition of phosphoinositide 3-kinase signaling in human breast cancer cells. Mol Cancer Ther.

[33] Thuy MN, Kam JK, Lee GC, Tao PL, Ling DQ, Cheng M, Goh SK, Papachristos AJ, Shukla L, Wall KL, Smoll NR, Jones JJ, Gikenye N (2015). A novel literature-based approach to identify genetic and molecular predictors of survival in glioblastoma multiforme: analysis of 14,678 patients using systematic review and meta-analytical tools. J Clin Neurosci.

[34] Bussink J, van der Kogel AJ, Kaanders JH (2008). Activation of the PI3-K/AKT pathway and implications for radioresistance mechanisms in head and neck cancer. Lancet Oncol.

[35] Li HF, Kim JS, Waldman T (2009). Radiation-induced Akt activation modulates radioresistance in human glioblastoma cells. Radiat Oncol.

[36] Kim IA, Bae SS, Fernandes A, Wu J, Muschel RJ, McKenna WG, Birnbaum MJ, Bernhard EJ (2005). Selective inhibition of Ras, phosphoinositide 3 kinase, and Akt isoforms increases the radiosensitivity of human carcinoma cell lines. Cancer Res.

[37] Kuger S, Graus D, Brendtke R, Gunther N, Katzer A, Lutyj P, Polat B, Chatterjee M, Sukhorukov VL, Flentje M, Djuzenova CS (2013). Radiosensitization of glioblastoma cell lines by the dual PI3K and mTOR inhibitor NVP-BEZ235 depends on drug-irradiation schedule. Transl Oncol.

[38] Baumann P, Mandl-Weber S, Oduncu F, Schmidmaier R (2009). The novel orally bioavailable inhibitor of phosphoinositol-3-kinase and mammalian target of rapamycin, NVP-BEZ235, inhibits growth and proliferation in multiple myeloma. Exp Cell Res.

[39] el-Deiry WS, Tokino T, Velculescu VE, Levy DB, Parsons R, Trent JM, Lin D, Mercer WE, Kinzler KW, Vogelstein B (1993). WAF1, a potential mediator of p53 tumor suppression. Cell.

[40] Hartwell LH, Kastan MB (1994). Cell cycle control and cancer. Science.

[41] Bunz F, Dutriaux A, Lengauer C, Waldman T, Zhou S, Brown JP, Sedivy JM, Kinzler KW, Vogelstein B (1998). Requirement for p53 and p21 to sustain G2 arrest after DNA damage. Science.

[42] Hazawa M, Yasuda T, Noshiro K, Saotome-Nakamura A, Fukuzaki T, Michikawa Y, Gotoh T, Tajima K (2012). Vitronectin improves cell survival after radiation injury in human umbilical vein endothelial cells. FEBS Open Bio.

[43] Czernin J, Weber WA, Herschman HR (2006). Molecular imaging in the development of cancer therapeutics. Annu Rev Med.

[44] Glunde K, Bhujwalla ZM (2011). Metabolic tumor imaging using magnetic resonance spectroscopy. Semin Oncol.

[45] Murphy PS, Viviers L, Abson C, Rowland IJ, Brada M, Leach MO, Dzik-Jurasz AS (2004). Monitoring temozolomide treatment of low-grade glioma with proton magnetic resonance spectroscopy. Br J Cancer.

[46] Venkatesh HS, Chaumeil MM, Ward CS, Haas-Kogan DA, James CD, Ronen SM (2012). Reduced phosphocholine and hyperpolarized lactate provide magnetic resonance biomarkers of PI3K/Akt/mTOR inhibition in glioblastoma. Neuro Oncol.

[47] Su JS, Woods SM, Ronen SM (2012). Metabolic consequences of treatment with AKT inhibitor perifosine in breast cancer cells. NMR Biomed.

[48] Sturm D, Bender S, Jones DT, Lichter P, Grill J, Becher O, Hawkins C, Majewski J, Jones C, Costello JF, Iavarone A, Aldape K, Brennan CW (2014). Paediatric and adult glioblastoma: multiform (epi)genomic culprits emerge. Nat Rev Cancer.

[49] Jones C, Baker SJ (2014). Unique genetic and epigenetic mechanisms driving paediatric diffuse high-grade glioma. Nat Rev Cancer.

[50] Liu TJ, Koul D, LaFortune T, Tiao N, Shen RJ, Maira SM, Garcia-Echevrria C, Yung WK (2009). NVP-BEZ235, a novel dual phosphatidylinositol 3-kinase/mammalian target of rapamycin inhibitor, elicits multifaceted antitumor activities in human gliomas. Mol Cancer Ther.

[51] Sjobakk TE, Johansen R, Bathen TF, Sonnewald U, Kvistad KA, Lundgren S, Gribbestad IS (2007). Metabolic profiling of human brain metastases using in vivo proton MR spectroscopy at 3T. BMC Cancer.

[52] Kwock L, Smith JK, Castillo M, Ewend MG, Collichio F, Morris DE, Bouldin TW, Cush S (2006). Clinical role of proton magnetic resonance spectroscopy in oncology: brain, breast, and prostate cancer. Lancet Oncol.

[53] Glunde K, Bhujwalla ZM, Ronen SM (2011). Choline metabolism in malignant transformation. Nat Rev Cancer.

[54] Liao EC, Hsu YT, Chuah QY, Lee YJ, Hu JY, Huang TC, Yang PM, Chiu SJ (2014). Radiation induces senescence and a bystander effect through metabolic alterations. Cell Death Dis.

[55] Bhatt AN, Chauhan A, Khanna S, Rai Y, Singh S, Soni R, Kalra N, Dwarakanath BS (2015). Transient elevation of glycolysis confers radio-resistance by facilitating DNA repair in cells. BMC Cancer.

[56] Al-Saffar NM, Troy H, Ramirez de Molina A, Jackson LE, Madhu B, Griffiths JR, Leach MO, Workman P, Lacal JC, Judson IR, Chung YL (2006). Noninvasive magnetic resonance spectroscopic pharmacodynamic markers of the choline kinase inhibitor MN58b in human carcinoma models. Cancer Res.

[57] Workman P, Aboagye EO, Balkwill F, Balmain A, Bruder G, Chaplin DJ, Double JA, Everitt J, Farningham DA, Glennie MJ, Kelland LR, Robinson V, Stratford IJ (2010). Guidelines for the welfare and use of animals in cancer research. Br J Cancer.

[58] Gowan SM, Hardcastle A, Hallsworth AE, Valenti MR, Hunter LJ, AK de Haven Brandon, Garrett MD, Raynaud F, Workman P, Aherne W, Eccles SA (2007). Application of meso scale technology for the measurement of phosphoproteins in human tumor xenografts. Assay Drug Dev Technol.

