# Supplementary material for: Pediatric and adult glioblastoma radiosensitization induced by PI3K/mTOR inhibition causes early metabolic alterations detected by nuclear magnetic resonance spectroscopy
Source: Oncotarget. 2017 May 24;8(29):47969–83. doi: 10.18632/oncotarget.18206 (PMC5564619; doi:10.18632/oncotarget.18206)
Supplement: Supplementary file 1 [file oncotarget-08-47969-s001.pdf]

Pediatric and adult glioblastoma radiosensitization induced by PI3K/mTOR inhibition causes early metabolic alterations detected by nuclear magnetic resonance spectroscopy

SUPPLEMENTARY MATERIALS

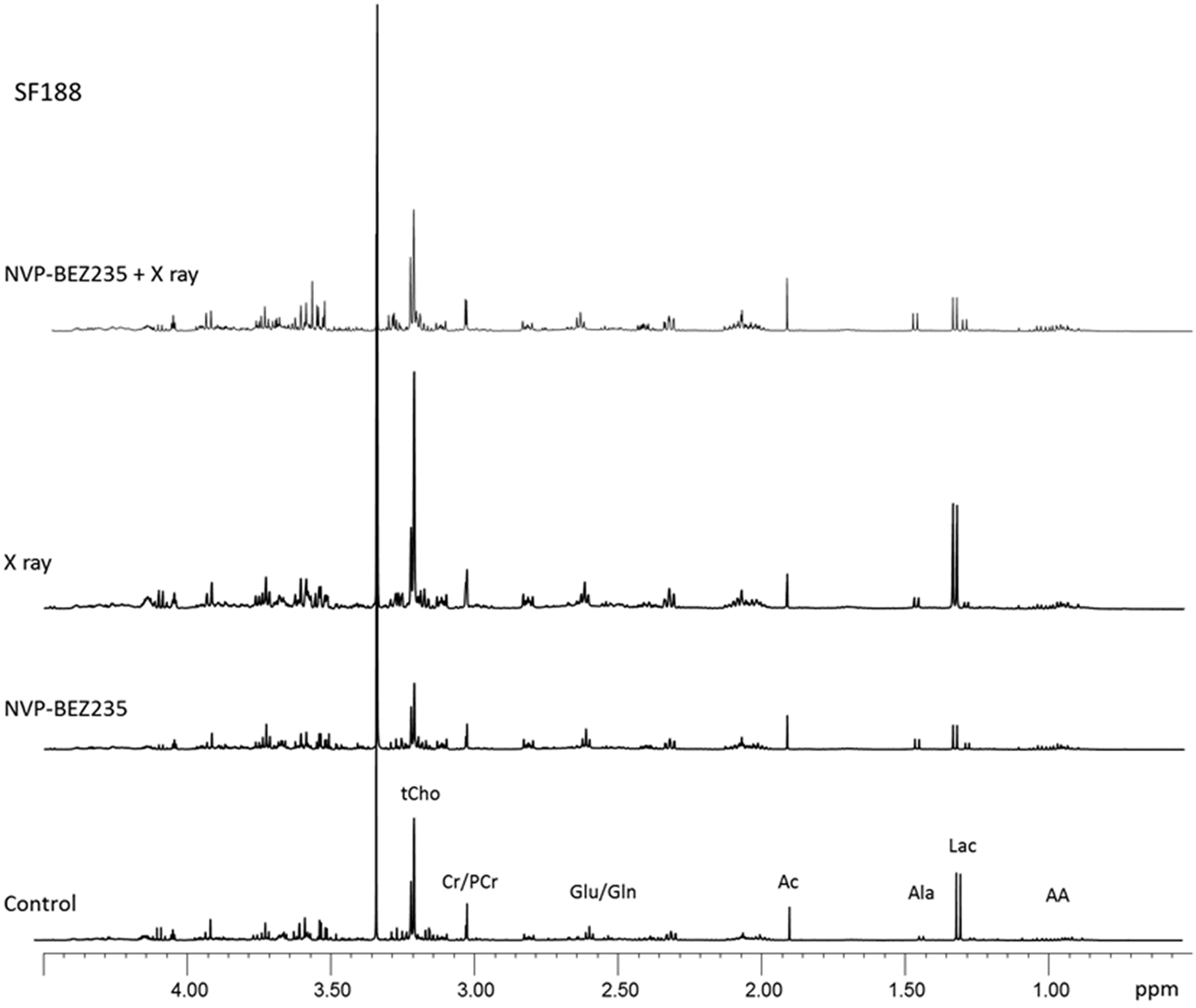

Supplementary Figure 1: Representative ^{1}H-NMR spectrum from SF188 cell extracts before or after treatment with NVP-BEZ235, X ray, alone or in combination. AA: aminoacids; Lac: lactate; Ala: alanine; Ac: acetate; Glu/Gln: glutamate/glutamine; Cr/PCr: creatine/phosphocreatine; tCho: total choline.

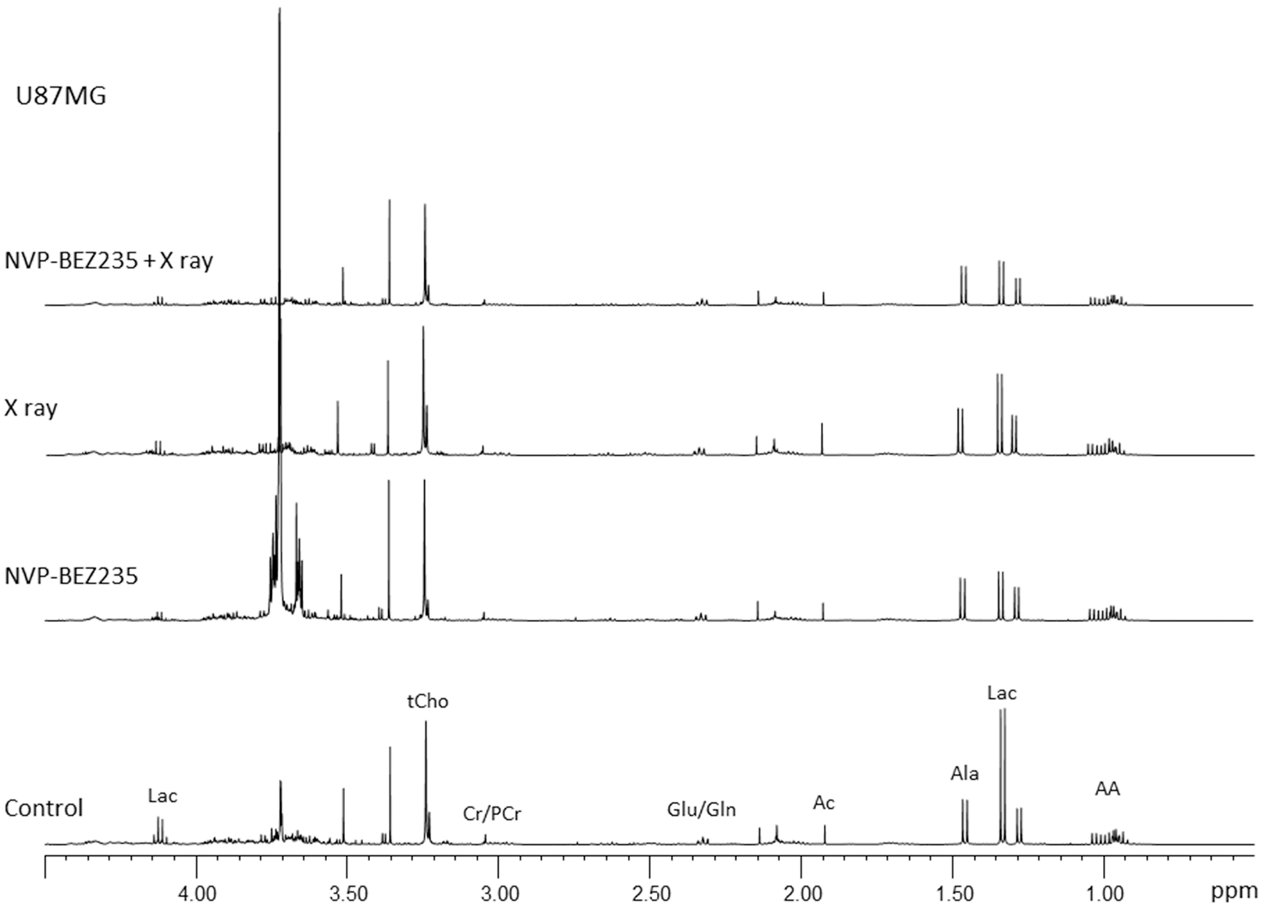

Supplementary Figure 2: Representative ^{1}H-NMR spectrum from U87MG cell extracts before or after treatment with NVP-BEZ235, X ray, alone or in combination. AA: aminoacids; Lac: lactate; Ala: alanine; Ac: acetate; Glu/Gln: glutamate/ glutamine; Cr/PCr: creatine/phosphocreatine; tCho: total choline.

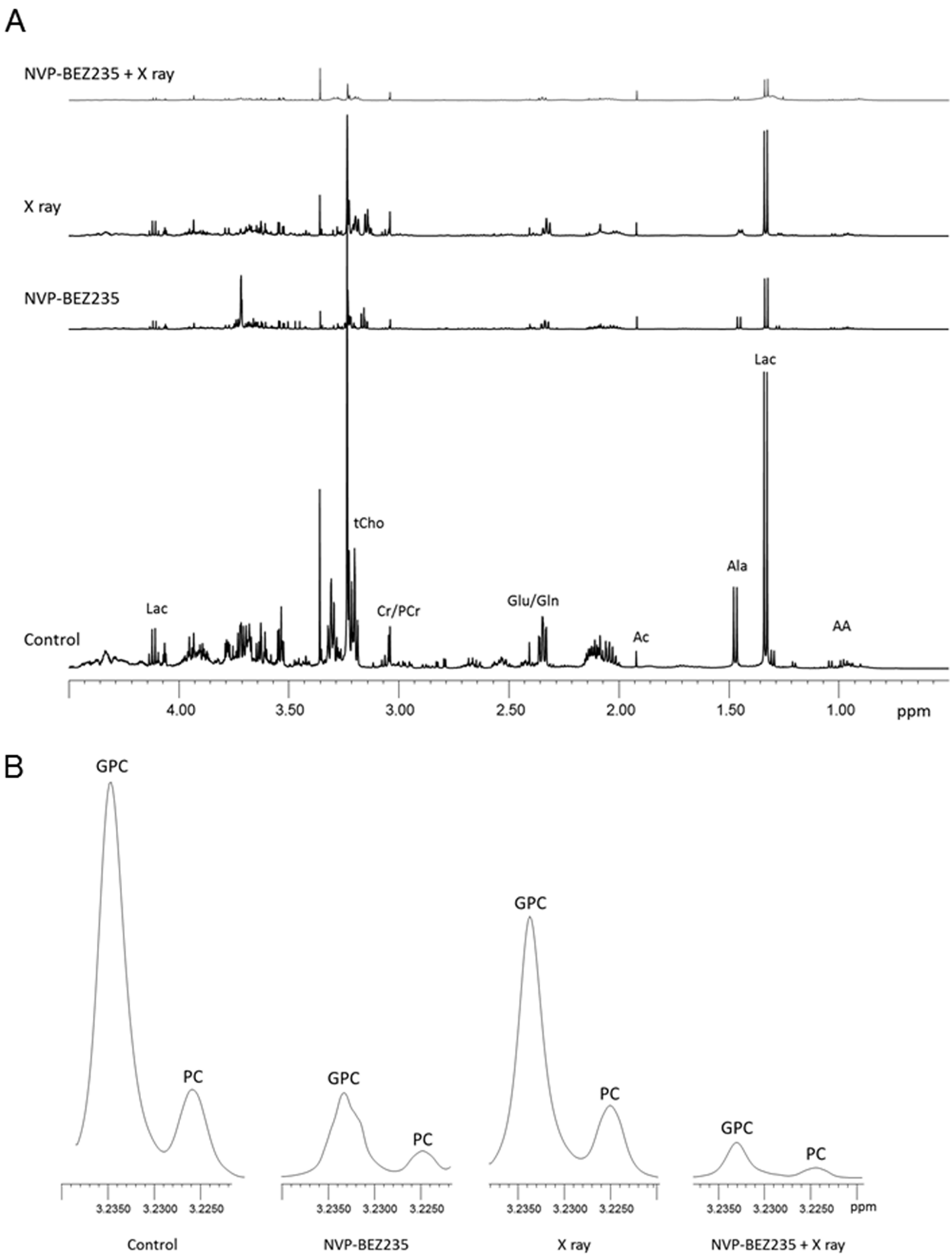

Supplementary Figure 3: (A) Representative ^{1}H-NMR spectrum from an ex vivo control U87MG tumor tissue extracts before or after treatment with NVP-BEZ235, X ray, alone or in combination. AA: aminoacids; Lac: lactate; Ala: alanine; Ac: acetate; Glu/Gln: glutamate/glutamine; Cr/PCr: creatine/phosphocreatine; tCho: total choline. **(B)** Representative zoom in of the choline area for control and treated spectra from an *ex vivo* U87MG tumor tissue extracts.

Supplementary Table 1: Densitometric analyses of immunoblots from SF188 cell extracts

See Supplementary File 1

Supplementary Table 2: Densitometric analyses of immunoblots from U87MG cell extracts

See Supplementary File 2

Supplementary Table 3: Densitometric analyses of immunoblots from U87MG xenograft tumors

See Supplementary File 3

Supplementary Table 4: Quantitative content of metabolites and PC/GPC ratio in control SF188 and U87MG cell lines

See Supplementary File 4
