# Supplementary material for: Pediatric and adult glioblastoma radiosensitization induced by PI3K/mTOR inhibition causes early metabolic alterations detected by nuclear magnetic resonance spectroscopy
Source: Oncotarget. 2017 May 24;8(29):47969–83. doi: 10.18632/oncotarget.18206 (PMC5564619; doi:10.18632/oncotarget.18206)
Supplement: Supplementary file 2 [file oncotarget-08-47969-s002.docx]

**Supplementary Table 1: Densitometric analyses of immunoblots from U87MG xenograft tumours**

|  | **NVP-BEZ235** | | | **Irradiation** | | | **NVP-BEZ235 + irradiation** | | |
| --- | --- | --- | --- | --- | --- | --- | --- | --- | --- |
|  | Average^*^ | SD | p | Average^*^ | SD | p | Average^*^ | SD | p |
| **pAKT^Ser473^/tot Akt** | 0.51 | 0.27 | 0.0448 | 0.98 | 0.16 | NS | 0.77 | 0.24 | NS |
| **pRPS6^Ser240/244^/tot RPS6** | 0.032 | 0.03 | 0.032 | 1.2 | 0.48 | NS | 0.036 | 0.02 | 0.032 |
| **CHKA** | 0.51 | 0.21 | 0.0128 | 1.23 | 0.15 | NS | 0.6 | 0.22 | 0.0368 |
| **HK2** | 0.69 | 0.35 | NS | 1.13 | 0.31 | NS | 0.53 | 0.24 | 0.0245 |
| *Fold change compared to the control | | | |  |  |  |  |  |  |

**ST1**: Arrows show significant change in protein levels compared to the control (n => 6).
