# Supplementary material for: Pediatric and adult glioblastoma radiosensitization induced by PI3K/mTOR inhibition causes early metabolic alterations detected by nuclear magnetic resonance spectroscopy
Source: Oncotarget. 2017 May 24;8(29):47969–83. doi: 10.18632/oncotarget.18206 (PMC5564619; doi:10.18632/oncotarget.18206)
Supplement: Supplementary file 3 [file oncotarget-08-47969-s003.docx]

**Supplementary Table 2: Densitometric analyses of immunoblots from U87MG cell extracts**

|  | **NVP-BEZ235** | | | **Irradiation** | | | **NVP-BEZ235 + irradiation** | | |
| --- | --- | --- | --- | --- | --- | --- | --- | --- | --- |
|  | Average^*^ | SD | p | Average^*^ | SD | p | Average^*^ | SD | p |
| **pAKT^Ser473^/tot Akt** | 0.48 | 0.43 | NS | 0.8 | 0.41 | NS | 0.19 | 0.16 | 0.0294 |
| **pRPS6^Ser240/244^/tot RPS6** | 0.66 | 0.19 | NS | 1.15 | 0.53 | NS | 0.079 | 0.02 | 0.0039 |
| **CHKA** | 0.92 | 0.25 | 0.0128 | 0.84 | 0.28 | NS | 0.42 | 0.34 | 0.033 |
| **HK2** | 0.94 | 0.14 | NS | 0.72 | 0.23 | NS | 0.48 | 0.18 | 0.0113 |
| *Fold change compared to the control | | | |  |  |  |  |  |  |

**ST2**: Arrows show significant change in protein levels compared to the control (n => 6).
