# Supplementary material for: Pediatric and adult glioblastoma radiosensitization induced by PI3K/mTOR inhibition causes early metabolic alterations detected by nuclear magnetic resonance spectroscopy
Source: Oncotarget. 2017 May 24;8(29):47969–83. doi: 10.18632/oncotarget.18206 (PMC5564619; doi:10.18632/oncotarget.18206)
Supplement: Supplementary file 4 [file oncotarget-08-47969-s004.docx]

**Supplementary Table 3: Densitometric analyses of immunoblots from U87MG xenograft tumours**

|  | **NVP-BEZ235** | | | **Irradiation** | | | **NVP-BEZ235 + irradiation** | | |
| --- | --- | --- | --- | --- | --- | --- | --- | --- | --- |
|  | Average^*^ | SD | p | Average^*^ | SD | p | Average^*^ | SD | p |
| **Cleaved PARP/PARP** | 7.6 | 16.0 | 0.3 | 2.9 | 5.9 | 0.4 | 6.6 | 5.4 | 0.03 |
| **LDHA** | 1.4 | 0.5 | 0.08 | 1.2 | 0.2 | 0.006 | 0.5 | 0.3 | 0.005 |
| **CHKA** | 0.3 | 0.5 | 0.0002 | 1.7 | 1.0 | 0.01 | 0.4 | 0.7 | 0.05 |
| *Fold change compared to the control | | | |  |  |  |  |  |  |

**ST3**: Arrows show significant change in protein levels compared to the control (n => 6).
