# Supplementary material for: Pediatric and adult glioblastoma radiosensitization induced by PI3K/mTOR inhibition causes early metabolic alterations detected by nuclear magnetic resonance spectroscopy
Source: Oncotarget. 2017 May 24;8(29):47969–83. doi: 10.18632/oncotarget.18206 (PMC5564619; doi:10.18632/oncotarget.18206)
Supplement: Supplementary file 5 [file oncotarget-08-47969-s005.docx]

**Supplementary Table 4: Quantitative content of metabolites and PC/GPC ratio in control SF188 and U87MG cell lines**

| **SF188 metabolite** | **Average _(µmoles/cell number)_** | **SD** |  |
| --- | --- | --- | --- |
| *Lactate* | 3.27 | 2.44 |  |
| *PC* | 0.83 | 0.37 |  |
| *GPC* | 0.43 | 0.2 |  |
| *Tot Choline* | 1.38 | 0.67 |  |
| *PC/GPC* | 1.96 | 0.14 |  |
| **U87MG metabolite** | **Average _(µmoles/cell number)_** | **SD** | **p** |
| *Lactate* | 1.98 | 0.84 | 0.022 |
| *PC* | 0.35 | 0.18 | 0.064 |
| *GPC* | 0.74 | 0.18 | 0.55 |
| *Tot choline* | 1.06 | 0.24 | 0.044 |
| *PC/GPC* | 0.52 | 0.27 | 0.0003 |
